# Reach of Messages in a Dental Twitter Network: Cohort Study Examining User Popularity, Communication Pattern, and Network Structure

**DOI:** 10.2196/10781

**Published:** 2018-09-13

**Authors:** Maha El Tantawi, Asim Al-Ansari, Abdulelah AlSubaie, Amr Fathy, Nourhan M Aly, Amira S Mohamed

**Affiliations:** 1 Department of Preventive Dental Sciences College of Dentistry Imam Abdulrahman Bin Faisal University Dammam Saudi Arabia; 2 Program of Computer and Communications Engineering Faculty of Engineering Alexandria University Alexandria Egypt; 3 Department of Pediatric Dentistry and Dental Public Health Faculty of Dentistry Alexandria University Alexandria Egypt

**Keywords:** social media, health communication, dentists, students, dental, social network analysis, twitter, social networks

## Abstract

**Background:**

Increasing the reach of messages disseminated through Twitter promotes the success of Twitter-based health education campaigns.

**Objective:**

This study aimed to identify factors associated with reach in a dental Twitter network (1) initially and (2) sustainably at individual and network levels.

**Methods:**

We used instructors’ and students’ Twitter usernames from a Saudi dental school in 2016-2017 and applied Gephi (a social network analysis tool) and social media analytics to calculate user and network metrics. Content analysis was performed to identify users disseminating oral health information. The study outcomes were reach at baseline and sustainably over 1.5 years. The explanatory variables were indicators of popularity (number of followers, likes, tweets retweeted by others), communication pattern (number of tweets, retweets, replies, tweeting/ retweeting oral health information or not). Multiple logistic regression models were used to investigate associations.

**Results:**

Among dental users, 31.8% had reach at baseline and 62.9% at the end of the study, reaching a total of 749,923 and dropping to 37,169 users at the end. At an individual level, reach was associated with the number of followers (baseline: odds ratio, OR=1.003, 95% CI=1.001-1.005 and sustainability: OR=1.002, 95% CI=1.0001-1.003), likes (baseline: OR=1.001, 95% CI=1.0001-1.002 and sustainability: OR=1.0031, 95% CI=1.0003-1.002), and replies (baseline: OR=1.02, 95% CI=1.005-1.04 and sustainability: OR=1.02, 95% CI=1.004-1.03). At the network level, users with the least followers, tweets, retweets, and replies had the greatest reach.

**Conclusions:**

Reach was reduced by time. Factors increasing reach at the user level had different impact at the network level. More than one strategy is needed to maximize reach.

## Introduction

Social media can reach people connected to the internet [1] at low cost [2,3] regardless of education or access to health care [4]. Twitter differs from other social media in the pattern of communication it supports. On Twitter, person A may follow person B without person B following back, or they may follow each other. This differs from Facebook where friendship is mutual [5]. Another difference is reciprocity which signifies the symmetry of social ties and is associated with higher connectivity [6]. Twitter has a low level of reciprocity, with only 22% of all connections reciprocated [5]. Twitter is also characterized by a hierarchical structure [6] where few popular users have a large number of followers and act as information brokers [7] disseminating information to the bulk of the Twitter network. Thus, Twitter plays 2 roles: (1) news media outlet spreading information in a one-to-many mode with most users on the receiving side and (2) social networking site where people connect and interact in a one-to-one communication pattern [8]. Differences by country and culture were reported regarding which pattern prevails, and this may affect the number of users reached in a network [6].

Also, connected Twitter users are included in a network. Some networks are more efficient at spreading information than others because of their structure such as small world networks. Social network analysis provides the tools to visualize this network and calculate metrics that characterize it and quantify users’ connectivity [5,8].

Social media has the potential to improve health by spreading health information [4]. Twitter was used to disseminate health information through accounts of ministries of health [9,10] professional associations [7] and local health departments [4]. There are fewer reports about using Twitter in dentistry including investigating the use of Twitter to share information among dental clinicians [11], modeling the impact of third molar experience on quality of life on Twitter [12] and describing antifluoridation activity dominance on social media [13]. Despite the increasing use of Twitter for health information purposes [14], little is known about the spread of health information on it [15]. This is important for planning Twitter-based public health interventions [10].

Saudi Arabia has the highest number of Twitter users among Arab countries (50% of Arab users and 47% of tweets) [16]. It offers an appropriate setting to investigate factors affecting tweet reach. The present report studies a Twitter network of instructors and students in a dental school and others connected to them. Potentially, oral health information can be disseminated by multiple users through different accounts in such a network. This differs from previous studies where there were campaigns with planned health messages generated by one account representing a health organization/ professional body [4,7,9,10,14].

In this study we hypothesize that tweet reach is affected by user popularity (followers, likes and retweets), communication pattern (tweeting, retweeting, replying and disseminating oral health information), and Twitter network characteristics. This study aimed to assess (1) factors associated with tweet reach at one point in time and (2) sustainably over time at the individual user and network levels.

## Methods

### Study Parameters

We conducted a cohort study of a Twitter network of instructors and students in the College of Dentistry, Imam Abdulrahman Bin Faisal University, Saudi Arabia. This governmental school offers a 6-year Bachelor of Dental Surgery program and had 58 instructors and 301 students in 2016-17. We followed the accounts from June 2016 to November 2017. The network included dental users who tweeted general oral health information topics in addition to other topics such as entertainment, sports, and academic life. They tweeted on their own (ad libitum) without following a specific agenda or any instructions. Their tweeting was, therefore, not standardized. Instead, it reflected their tweeting habits. It also included nondental users who follow them. Ethical approval was obtained from the research unit at the College of Dentistry (#EA 2014009).

### Study Population

Participants were included if (1) they were affiliated with the college (dental users) in June 2016 and (2) had an open Twitter account. Lists of participants were obtained from academic affairs. They were contacted, introduced to the study and asked for their Twitter usernames. Consent was not sought from them since their accounts were open [17].

The Twitter network was constructed using a software pipeline (Multimedia Appendix 1: Software pipeline), the open-source graph visualization platform Gephi [20] and social network analysis [21]. Metrics of network characteristics were calculated including [5,8,21]:

1. Number of users and connections between them.
2. Number of connected components: if two users are linked directly or indirectly through a third user, they are in a connected component.
3. The average path length: the number of users needed for A to connect to B is the path length with shorter paths helping information spread.
4. Network diameter: the longest distance between two users is the network diameter.
5. Degree: the number of connections a user has is the degree. Its probability distribution shows the type of network.

We used Twitter accounts dashboards to obtain the number of followers, tweets, and likes. Users were categorized into those with the least number of followers (≤200) [8], those with the highest number of followers (≥1000), and those with a moderate number of followers in between. We used twitonomy [22] to obtain the number of replies, retweets, and tweets retweeted by others. We categorized the number of tweets, retweets, and replies into high and low levels using their respective 75th percentile as cutoff points. Tweets and retweets from January to June 2016 were used to identify users who are tweeting or retweeting oral health information. To calibrate, 2 investigators manually coded [19] the messages of 20 users employing content analysis and differences were resolved by discussion until an acceptable agreement (kappa=.7) was established.

### Statistical Analysis

Univariate and multiple logistic regression models were developed for 2 binary outcome variables: (1) reach at baseline, and (2) sustained reach. The explanatory variables were: (1) indicators of user popularity (number of followers, likes and retweeted tweets) and (2) communication pattern (number of tweets, retweets, replies, and whether the user tweeted or retweeted oral health information). All variables were entered into the multiple model so that they were adjusted for each other, gender, and role (instructor/student). Significance was set at the 5% level. Statistical analysis was performed using SPSS version 22.0 (IBM Corp., Armonk, N.Y., USA).

## Results

Thirty-nine of 58 (67.2%) instructors and 225/301 (74.8%) students responded. Table 1 shows that most dental users were students (225/264, 85.2%) and males (146, 55.3%). The median number of followers was 170 with an interquartile range (IQR) of 69-340 with a median of 81 likes (IQR 14-454) and 29 tweets (IQR 6-118) retweeted. Of the 1.1 million tweets generated, 1.7% were retweets and 0.8% were replies. Oral health information was equally tweeted (20, 7.6%) and retweeted (21, 8.0%).

Of all dental users, 84 (31.8%) had reach at baseline and 166 (62.9%) at the end of the study. The median number reached at baseline was 0 (IQR 0-4) increasing at the end to a median of 4 (IQR 0-211). The total number reached at baseline was 749,923 dropping to 37,169 at the end (95.0% reduction). There were 71 (26.9%) dental users with sustained reach.

The results of the univariate logistic regression are shown in Multimedia Appendix 2. Table 2 shows that users with more followers had significantly higher odds of reaching people at baseline with an odds ratio (OR)=1.003, 95% CI=1.001-1.005 and sustainably (OR=1.002, 95% CI=1.0001-1.003). Those with more likes (baseline: OR=1.001, 95% CI=1.0001-1.002 and sustainability: OR=1.001, 95% CI=1.0003-1.002) and with more replies (baseline: OR=1.02, 95% CI=1.005-1.04 and sustainability: OR=1.02, 95% CI=1.004-1.03) had significantly higher odds of reaching others. Tweeting oral health information was associated with significantly higher odds of reach at baseline (OR=5.07, 95% CI=1.18-21.69) but had no significant association with sustained reach (OR=2.99, 95% CI=0.77-11.53).

**Figure 1:**
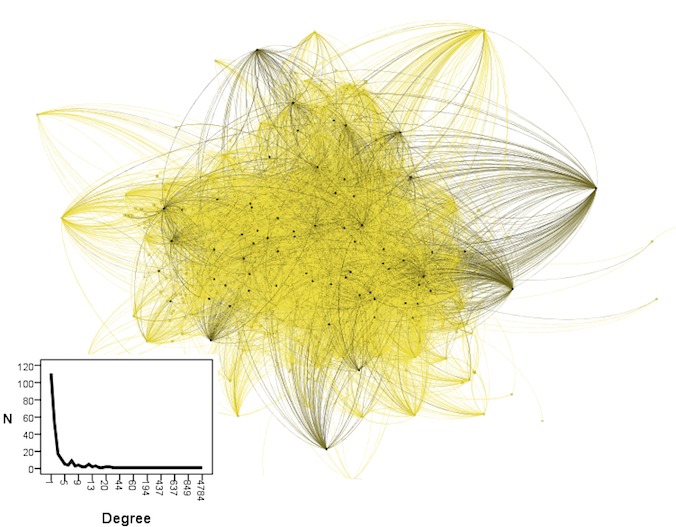
Twitter network, black nodes are dental users, yellow nodes are nondental users with a power-law distribution of degrees in the inset.

**Figure 2:**
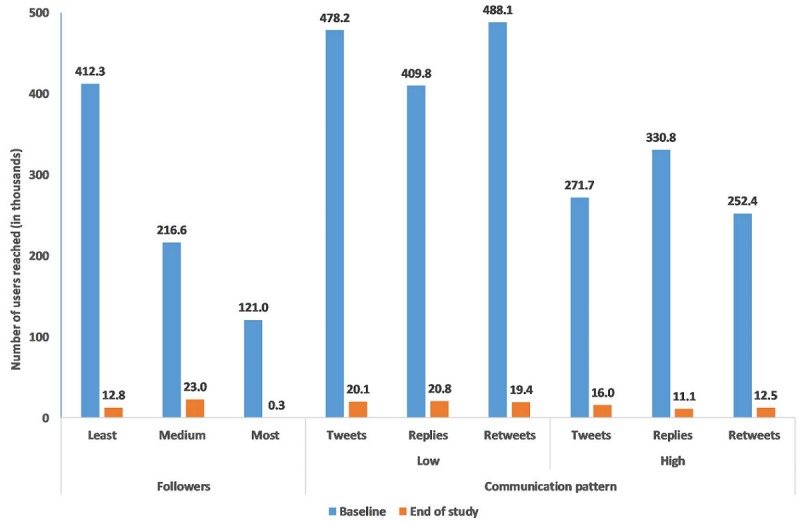
Reach at different time points for users with different followers levels and communication patterns.

There were 264 dental users and 46,951 nondental users for a total of 47,215 users and 77,309 connections (Figure 1). The network diameter was 9 with an average path length of 4.253 and 3 connected components the largest of which included 99.9% of all users. Dental users (represented by the black nodes) were a minority with nondental users (yellow nodes) forming the majority of the network. The inset inside Figure 1 shows that the users’ degrees had a power law distribution which is characteristic of small world networks. Figure 2 shows that collectively, dental users with the least number of followers had >3 times the reach of users with the highest number of followers. Users with low number of tweets, replies and retweets had greater reach than those with high levels of these messages.

**Table 1:** Dental users’ characteristics and communication pattern in a dental school Twitter network involving instructors (N=39) and students (N=225).

Factors			Analysis, n (%)
**User characteristics**			
	**Role**		
		Instructor	39 (14.8)
		Student	225 (85.2)
	**Gender**		
		Male	146 (55.3)
		Female	118 (44.7)
	**Number of followers**		
		Median (IQR^a^)	170 (69-340)
		Total	82,011
		≤200	146 (55.3)
		201-999	105 (39.8)
		≥1000	13 (4.9)
	**Number of likes**		
		Median (IQR)	81 (14-454)
		Total	123,473
	**Number of tweets retweeted by others**		
		Median (IQR)	29 (6-118)
		Total	22,927
**Communication pattern**			
	**Number of tweets**		
		Median (IQR)	1,114 (180-5,089)
		Total	1,116,225
	**Number of retweets**		
		Median (IQR)	23 (5-94)
		Total (% of all tweets)	19,015 (1.7)
	**Number of replies**		
		Median (IQR)	8 (1-37.3)
		Total (% of all tweets)	8,748 (0.8)
Tweeted oral health information			20 (7.6)
Retweeted oral health information			21 (8.0)

^a^IQR: interquartile range.

**Table 2:** Factors associated with having reach at baseline and sustained reach.

Reach factors			Multiple logistic regression	
			Adjusted odds ratio^a^ (95% CI)	*P* value
**Having reach at baseline**				
	**Indicators of user popularity**			
		Number of followers	1.003 (1.001-1.005)^b^	.003^c^
		Number of likes	1.001 (1.0001-1.002)^b^	.03^c^
		Number of tweets retweeted by others	0.98 (0.95-1.00)	.10
	**Communication pattern**			
		Number of tweets	1.00 (1,00-1.00)	.18
		Number of tweets that are retweets	1.02 (1.00-1.06)	.11
		Number of tweets that are replies	1.02 (1.005-1.04)^b^	.009^c^
Tweeted oral health information versus not			5.07 (1.18-21.69)^b^	.03^c^
Retweeted oral health information versus not			0.66 (0.13-3.24)	.57
**Having sustained reach**				
	**Indicators of user popularity**			
		Number of followers	1.002 (1.0001-1.003)^b^	.01^c^
		Number of likes	1.001 (1.0003-1.002)^b^	.02^c^
		Number of tweets retweeted by others	0.98 (0.95-1.01)	.17
	**Communication pattern**			
		Number of tweets	1.00 (1.00-1.00)	.45
		Number of tweets that are retweets	1.02 (0.99-1.05)	.17
		Number of tweets that are replies	1.02 (1.004-1.03)^b^	.01^c^
Tweeted oral health information versus not			2.99 (0.77-11.53)	.11
Retweeted oral health information versus not			0.83 (0.17-3.95)	.82

^a^Controlling for all variables in addition to role (instructor/student) and gender.

^b^Statistically significant CI not including null value.

^c^Statistically significant *P*<.05.

## Discussion

### Principal Findings

The present study showed that the Twitter network structure facilitated information spread and that user popularity and communication pattern significantly but differently affected reach at individual and network levels. At the individual level, popular users and a communication pattern with replies had higher odds of reach. However, at the network level, users with the least number of followers and those with lower levels of tweets, retweets and replies collectively reached more users. Thus, our results support the study hypothesis.

At the individual user level, more followers increased the odds of reach. This reflects the emergence of the phenomenon of social media influencers [23] who affect public opinion because they can reach many users. Our results agree with a study [8] showing that few users with a large number of followers can spread information to a large portion of a Twitter network. It disagrees, however, with another study [7] showing that the Twitter accounts of 4 medical associations had large numbers of followers ranging from 6-213 thousand users, but their actual reach and information dissemination level was low. The difference between our study and theirs may be attributed to communication pattern and content of the message. The ORs for some variables are close to 1 indicating weak associations. For example, an increase of one follower or one like increases the chances that a user has reach by 0.1%-0.3%. However, the user has a median of 170 (interquartile range, IQR 69-340) followers and a median of 81 likes (IQR 14-454) resulting in a potentially greater effect per user. It is important to keep this scale perspective in mind for a better understanding of the impact of the variables.

In the present study, reach was positively affected by a two-way communication pattern with replies. This agrees with a previous study [24] indicating that replies were associated with greater message diffusion. Our results disagree with others indicating that replies address specific users, not the whole Twitter network so that those not involved might be disengaged [25]. User popularity and context of reply may explain the difference between the 2 sides. For example, replies from popular users may be more valued than others [26]. On the other hand, a corporate account replying to customers’ complaints may have low reach because users are driven off by the negative experiences discussed [26]. In the present study, the odds ratio for the impact of the number of followers on reach was reduced when the number of replies was included in the multiple model indicating possible effect modification since users with a large number of followers may have difficulty replying to others [8].

In the present study, the small portion of dental users disseminating oral health information reduced the possible impact of the network on patient education and the reason for this requires further investigation in future studies. Our analysis shows that tweeting oral health information increased the odds of reach at baseline but not on a sustained basis indicating that dental users who disseminate oral health information have greater reach than others. To keep this reach, they have to adopt additional measures to address the continuous demand for new topics on social media. The associations in the present study were observed in a Twitter network of dental professionals and their followers. Future studies are needed to assess the spread and reach of oral health information in a Twitter network of lay people and the factors affecting it.

The present study showed that at the network level, less popular users and those with lower levels of tweets, retweets and replies collectively had the greatest reach. This can be explained by the theory of information flow [27] which indicates that users with a small number of followers; the grassroots, have a large presence in the network and because of this, they assume a marked role in information flow reflecting collective network intelligence [5]. Our results agree with a report [28] showing that Twitter users with few followers were frequent sources and disseminators of Zika virus content on Twitter similar to public health institutions.

The Twitter network in the present study had features of small worlds which are reported to be efficient in spreading information. For example, a user was separated on average from others by 4 users. This agrees with a study [5] reporting on 41.7 million Twitter users where the average path length was 4.12 and with evidence showing an average path length on Facebook of 4.57 [29]. By contrast, Microsoft Network messenger had a greater path length (with median of 8) [5] suggesting differences among social media in supporting information spread. The vast majority of users in the present study were included in one large connected component allowing potential interaction among them. This agrees with statistics [8] reporting that 94.8% of Twitter users are included in 1 large connected component, and it is possible that this level of connectivity may be an inherent Twitter feature with potential for health education.

The number of users reached at baseline was drastically reduced by the end of the present study. This attrition is characteristic of social media-based experiences [30]. Twitter trends, characterized by spikes in the number of users discussing a specific topic, extend for a week or less with few lasting more than two months [5,10] indicating the duration people are expected to remain interested in 1 topic on Twitter. In the present study more than one user was involved and various topics were discussed, and reach was still attained after a longer period although at a much reduced level. Despite the drop in total reach, the number of dental users with reach and their median reach increased over the study period. This may be explained by the profile of new users joining the network. Studies are needed to investigate this phenomenon and how it affects the reach of the network.

It is difficult to directly compare the reach of several users sending unplanned messages in the present study with previous studies about Twitter campaigns/accounts of single users with planned messages. For example, a shisha campaign had 563 followers over 9 months after disseminating 373 tweets [31], and the Mayo Clinic account had 1,235 followers over 12 months after generating 1,635 tweets [32]. Our results show that the number of followers is critical to reach. Compared to the number of followers in these previous studies, the number in our study (median 170, IQR 69-340) indicates underused opportunity to spread oral health information.

### Limitations

The present study has some limitations. First, some dental users, who were not affiliated with the college such as practicing dentists or students in other dental schools, may have been classified as nondental users. Second, we calculated reach regardless of whom the user was and possibly including duplicate or corporate accounts. Such accounts would not benefit from oral health information and including them may overestimate reach. Third, we did not consider reach through other methods than following a user such as by hashtags [33], and this may underestimate reach. Our study has several strengths though. The Twitter network included users from a dental school, which increases the credibility of the information they spread through an inherent, nonformal peer review system. This large number of users and tweets and the long follow up period provide valid and realistic estimates about reach and factors affecting it. Further studies are needed to better understand the impact of a change in network structure and message content on reach.

### Conclusions

In a population of high Twitter use, a large number of non-dental users can be reached through Twitter with implications for health education. This potential impact is expected to increase as the percentage of users disseminating oral health information increases. Without intervention, a small portion of dental users would elect to do so indicating the need for incentives. If their involvement is recognized as part of community service activities, users from the academic dental sector may be encouraged to participate thus educating many more individuals that can be done in traditional health education. Multiple strategies are needed to maximize reach including the recruitment of popular users to disseminate oral health information, ensuring the presence of users who reply to inquiries and mobilizing grassroots to circulate messages through the network [5].

## Appendix

Multimedia Appendix 1 Software pipeline.

Multimedia Appendix 2 Factors associated with having reach at baseline and sustained reach (univariate logistic regression).

## Abbreviations

IQR: interquartile range
OR: odds ratio
