# Supplementary material for: Reach of Messages in a Dental Twitter Network: Cohort Study Examining User Popularity, Communication Pattern, and Network Structure
Source: J Med Internet Res. 2018 Sep 13;20(9):e10781. doi: 10.2196/10781 (PMC6231799; doi:10.2196/10781)
Supplement: Multimedia Appendix 1 [file jmir_v20i9e10781_app1.pdf]

Network construction

A directed graph G (V, E) is constructed where V is the set of vertices (nodes representing dental and non-dental users), and E is the set of edges based on the follow relationship between nodes in V. An edge (u, v) $\in$ E if and only if u follows v and u,v $\in$ V. The following steps were executed and they are represented in Figure 1:

1. The set of twitter usernames (dental users) obtained from faculty and students is specified in an external file readable by the software.
2. The Twitter Application Programming Interface (API) parameters' keys are stated in a configuration file in a simple key-value pair format to be accessible anywhere in the software pipeline.
3. A scraping tool on top of "Python Twitter Tools" was built (https://git.io/vbxHN). This is a minimalistic wrapper for the Python programming language around Twitter API to provide a user-friendly access to the original Twitter API.
    a. The tool starts by reading the usernames from the external file.
    b. A new object is created from the Twitter wrapper to be used later in the queries.
    c. For each username, data is fetched (following and followers lists) from the wrapper object. The API returns the user id, so the corresponding username must be looked up for an id.
    d. The fetched data is formatted and saved into an Excel sheet using the "openpyxl" Python library, which is a 3rd party software to create and manipulate Excel files.
4. A nodes list is generated by reading all the following and followers lists for all previously specified usernames. Duplicates are removed and then the new list is exported to an Excel sheet using "openpyxl".
5. Similarly, an edges matrix is generated where the username is the source and all the followings are targets. Then the sheets (for different usernames, as in #3.d above) are cross referenced for any relations.

6. The open-source graph visualization platform Gephi (https://gephi.org/) was used to represent the network and calculate its metrics.
    a. Nodes with degree <1 were filtered out to reduce noise and enable better visualization. The layout “Force Atlas2” was used to plot the network.

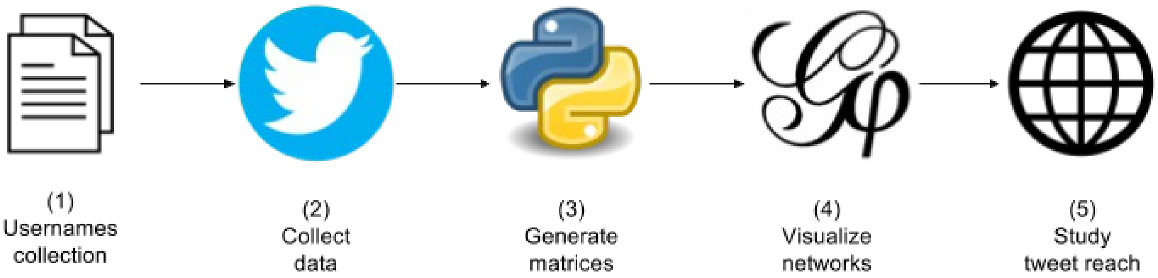

Figure 1: Software pipeline used to characterize the twitter network
