# Supplementary material for: Reach of Messages in a Dental Twitter Network: Cohort Study Examining User Popularity, Communication Pattern, and Network Structure
Source: J Med Internet Res. 2018 Sep 13;20(9):e10781. doi: 10.2196/10781 (PMC6231799; doi:10.2196/10781)
Supplement: Multimedia Appendix 2 [file jmir_v20i9e10781_app2.pdf]

Table 1. Factors associated with having reach at baseline and sustained reach (univariate logistic regression).

Reach factors	Univariate logistic regression	
	UOR[a] (95% CI)	*P* value
**Having reach at baseline**		
**Indicators of user popularity**		
No. of followers	1.004 (1.003-1.006)[b]	<.001[c]
No. of likes	1.001 (1.001-1.002)[b]	<.001[c]
No. of tweets retweeted by others	1.005 (1.003-1.008)[b]	<.001[c]
**Communication pattern**		
No. of tweets	1.0001 (1.00005-1.0002)[b]	<.001[c]
No. of tweets that are retweets	1.005 (1.003-1.008)[b]	<.001[c]
No. of tweets that are replies	1.02 (1.01-1.03)[b]	<.001[c]
Tweeted OHI[d] versus not	2.97 (1.17-7.54)[b]	.02[c]
Retweeted OHI[d] versus not	2.41 (0.97-5.95)	.06
**Having sustained reach**		
**Indicators of user popularity**		
No. of followers	1.003 (1.002-1.005)[b]	<.001[c]
No. of likes	1.0008 (1.0005-1.001)[b]	<.001[c]
No. of tweets retweeted by others	1.004 (1.002-1.006)[b]	<.001[c]
**Communication pattern**		
No. of tweets	1.00007 (1.00003-1.0001)[b]	.001[c]
No. of tweets that are retweets	1.004 (1.002-1.006)[b]	<.001[c]
No. of tweets that are replies	1.01 (1.006-1.02)[b]	<.001[c]
Tweeted OHI[d] versus not	2.30 (0.91-5.86)	.08
Retweeted OHI[d] versus not	2.03 (0.81-5.09)	.13

[a]UOR: unadjusted odds ratio.
[b]Statistically significant CI not including null value.
[c]Statistically significant *P*<.05.
[d]OHI: oral health information.
